# Glosario para una pandemia: el ABC de los conceptos sobre el coronavirus

**DOI:** 10.7705/biomedica.5605

**Published:** 2020-11-11

**Authors:** Felipe Botero-Rodríguez, Óscar H. Franco, Carlos Gómez-Restrepo

**Affiliations:** 1 Departamento de Epidemiología Clínica y Bioestadística, Facultad de Medicina, Pontificia Universidad Javeriana, Bogotá, D.C., Colombia Pontificia Universidad Javeriana Departamento de Epidemiología Clínica y Bioestadística Facultad de Medicina Pontificia Universidad Javeriana BogotáD.C. Colombia; 2 Institute of Social and Preventive Medicine, University of Bern, Bern, Switzerland University of Bern Institute of Social and Preventive Medicine University of Bern Bern Switzerland; 3 Departamento de Epidemiología Clínica y Bioestadística, Departamento de Psiquiatría y Salud Mental, Facultad de Medicina, Pontificia Universidad Javeriana, Bogotá, D.C., Colombia Pontificia Universidad Javeriana Departamento de Psiquiatría y Salud Mental Facultad de Medicina Pontificia Universidad Javeriana BogotáD.C Colombia; 4 Hospital Universitario San Ignacio, Pontificia Universidad Javeriana, Bogotá, D.C., Colombia Pontificia Universidad Javeriana Pontificia Universidad Javeriana BogotáD.C Colombia

**Keywords:** pandemia, infecciones por coronavirus, virus del SRAS, prevención y mitigación, descriptores de las ciencias de la salud, Pandemics, coronavirus infections, SARS virus, prevention and mitigation, medical subject headings

## Abstract

Actualmente, el mundo se enfrenta a la pandemia generada por el SARS-CoV-2, infección para la cual no hay medidas farmacológicas de prevención ni tratamiento. Hasta el momento, ha dejado más de 4'880.000 casos confirmados y 322.000 muertes. Se han propuesto diferentes estrategias para el control de la enfermedad que implican la participación de diferentes sectores de la sociedad con acciones guiadas por lineamientos jurídicos y basados en medidas de salud pública, entre ellas, la contención, la mitigación, el aislamiento físico y la cuarentena. Dado que se trata de una situación de dimensión poblacional, la información tiene un papel fundamental; sin embargo, la proliferación de términos nuevos, muchas veces usados erróneamente, causa confusión y desinformación y, en consecuencia, limitan la participación ciudanía.

En ese contexto, en el presente documento se hizo una revisión de los términos utilizados en epidemias y pandemias de enfermedades infecciosas, con énfasis en la COVID-19, para facilitar al público general la comprensión de los términos relevantes sobre el comportamiento de los agentes patógenos y de su ciclo epidémico y pandémico, así como los criterios para la adopción de las decisiones pertinentes en salud pública. Se aspira a que el glosario resultante ayude al uso correcto de los términos y a homogenizar la información.

## Abstract (en)

Currently, the world is facing the pandemic generated by SARS-CoV-2. There are no no pharmacological measures for the prevention or treatment of this infection and, so far, it has caused more than 4'880.000 confirmed cases and 322.000 deaths. The different strategies for the control of the disease that have been proposed involve the participation of different actors. Such participation, guided by legal guidelines based on public health measures, include containment, mitigation, physical isolation, and quarantine. As this is a population-based problem, information plays a primary role; however, the many new terms hat have arisen and their misuse confuse and, therefore, misinform thus limiting citizen participation. For this reason, we conducted a review of the terms used in epidemics and pandemics of infectious diseases, particularly COVID-19. We considered and differentiated the relevant terms to facilitate the understanding of pathogen's behavior and epidemic and pandemic cycles, as well as the criteria for public health decision-making for the general public. This glossary should facilitate the use of the terms and standardize the information.

Actualmente, el mundo se enfrenta a la enfermedad (COVID-19) causada por un beta coronavirus (SARS-CoV-2) cuya aparición fue reportada el 31 de diciembre del 2019 y que se diseminó rápidamente en todo el mundo a tal velocidad que el 11 de marzo de 2020 la Organización Mundial de la Salud la declaró pandemia ^1^^,^^2^. La magnitud de esta infección es comparable con la de la pandemia de influenza de 1918, de gran virulencia y letalidad; sin embargo, su transmisión ha sido más rápida y su alcance no se conoce completamente, pues todavía se encuentra en las fases incipientes de su desarrollo. En la pandemia de 1918 diversos factores de tipo económico, demográfico y cultural confluyeron para propiciar la instauración y diseminación de la infección, poniendo de relieve la importancia de la interacción entre las autoridades de salud y la población ^3^.

En el siglo XXI no habían aparecido pandemias de tal magnitud, no obstante, hubo grandes epidemias como la ocasionada por el síndrome respiratorio agudo grave, también originado en China en el 2002; el síndrome respiratorio del Oriente Medio (MERS) en el 2012, y la pandemia por el virus de influenza A (H_1_N_1_) en el 2009 ^4^^,^^5^. Hasta el 19 de mayo, la enfermedad causada por el virus SARS-CoV-2 había dejado más de 4'880.000 casos confirmados y 322.000 muertes ^6^. Dado que no se cuenta con vacunas ni con medidas farmacológicas para su prevención o tratamiento, se han propuesto diferentes estrategias para su control, las cuales implican acciones de los gobiernos, la comunidad y los individuos a nivel regional.

Entre estas acciones se destacan la contención, la mitigación, el aislamiento físico y la cuarentena, términos que pueden causar confusión y ser utilizados erróneamente, lo que limita la participación de la población en las medidas de control adoptadas. La participación de las comunidades implica el fortalecimiento de sus recursos propios, es decir, los conocimientos y estructuras organizacionales que garanticen su aporte real en los procesos de cambio requeridos para el control de las enfermedades infecciosas. En este sentido, la Organización Mundial de la Salud (OMS) propicia la participación y el empoderamiento de la población como estrategia fundamental en el manejo de la crisis actual y de las que eventualmente se den en el futuro ^7^.

En este contexto, se presentan una revisión y un glosario de los términos utilizados en las epidemias y pandemias de enfermedades infecciosas. Los términos se organizaron por secciones según los aspectos más relevantes durante una pandemia: 1) las bases para entender el comportamiento de un agente patógeno; 2) el ciclo de las epidemias y las pandemias; 3) los criterios que sustentan las decisiones en torno a las medidas de salud pública, y 4) las medidas de salud pública para enfrentar la pandemia.

## Metodología

Inicialmente, se propusieron términos que fueran pertinentes desde el punto de vista de las ciencias básicas, la salud pública y la epidemiología y, a medida que se encontraban nuevos conceptos, se incluían en el manuscrito.

La búsqueda de los términos se hizo en las bases de datos de literatura médica Medline y Scielo, en los sitios web de entidades líderes en el control de la pandemia como la OMS o el Ministerio de Salud y Protección Social de Colombia, y en textos sobre las áreas del conocimiento concernientes al tema. Posteriormente, se empleó una estrategia de bola de nieve para llegar a otras referencias y completar los conceptos. Por último, de manera inductiva se establecieron las categorías que se presentan a continuación para la exposición de los conceptos, inclyendo en cada una de ellas los términos por orden alfabético.

## Resultados

### Bases para entender el comportamiento de un agente patógeno

*ADN:* siglas del ácido desoxirribonucleico. Es el material hereditario de todos los organismos con excepción de los virus ARN. Está conformado por dos cadenas helicoidales polinucleótidas en forma de hélice ^8^.

*ARN:* siglas del ácido ribonucleico. Es una cadena polinucleótida individual en la que el azúcar ribosa reemplaza la desoxirribosa y el uracilo a la timina que se encuentran en las cadenas de ADN. Participa en la síntesis proteica ^8^. El SARS-CoV-2 tiene una única cadena de ARN ^9^.

*Bacteria:* organismo procariota unicelular vital para los ecosistemas del planeta y con capacidad de vivir en condiciones extremas. Existen múltiples especies con diferentes funciones, algunas de las cuales pueden causar enfermedades en humanos, siendo las más mortales las infecciones respiratorias como la tuberculosis ^8^.

*Brote:* aumento súbito de los casos de una enfermedad ^10^.

*Contagio:* transmisión de una enfermedad por contacto con el agente patógeno ^8^^,^^10^.

*Curva epidemiológica:* representación gráfica del número de casos epidémicos en una población. Sirve para detallar el patrón, la magnitud y la tendencia de la epidemia. En este sentido, se habla de "aplanamiento de la curva" es decir, el comportamiento de la curva epidemiológica cuando el número de casos activos se mantiene estable y constante en el tiempo, o sea, cuando disminuye la tasa de contagio ^10^.

*Enfermedad endémica:* enfermedad infecciosa que afecta de forma permanente o secular a las personas en un lugar determinado ^10^, por ejemplo, el dengue en zonas tropicales.

*Epidemia:* propagación rápida y anormal de una enfermedad infecciosa en un número superior al esperado ^10^.

*Fuente:* punto de origen del agente infeccioso, el cual puede llegar hasta el huésped susceptible (puede ser el reservorio) ^10^.

*Huésped:* organismo que alberga a otro en su interior, o que lo porta, por ejemplo, el sujeto afectado por una infección parasitaria ^10^.

*Número reproductivo* (R_0_): número promedio de infecciones resultantes de un solo caso en una población totalmente susceptible que no ha estado sujeta a intervención ^10^^,^^11^. En el caso de la COVID-19 se han calculado diferentes R_0_; una de las cifras iniciales en China fue de 2,2 (IC_95%_: 1,4-3,9), lo que significa que una persona infectada contagiaba a 2,2 personas más ^12^.

*Número reproductive efectivo* (R_t_): se puede considerar como la version en la "vida real" del R_0_. Es el número promedio de infecciones resultantes de un caso en una población susceptible cuando se están llevando a cabo intervenciones de prevención y control ^10^^,^^11^.

*Pandemia:* propagación mundial de una enfermedad generada por un microorganismo que se transmite de forma eficaz y es capaz de producir casos por transmision comunitaria en múltiples lugares ^2^.

*Parásito:* ser vivo que vive, se alimenta y crece dentro de un huésped; puede ser de parasitismo obligado, es decir, solamente vive en asociación con otro ser vivo, o facultativo, pues además de vivir como parásito, lo puede hacer de otras maneras ^8^.

*Patógeno:* agente biológico externo que se aloja en un organismo biológico determinado y causa infección. Puede ser de varios tipos: virus, bacteria, parásito u hongo ^8^^,^^10^.

*Periodo clínico:* lapso en el que aparecen los síntomas y los signos característicos de la enfermedad ^10^.

*Pico de curva:* punto más alto de una curva epidémica; representa el punto de mayor magnitud. En el caso de la COVID-19 será el momento en que la enfermedad alcance el mayor nivel de contagio e incidencia ^10^.

*Periodo de incubación:* intervalo de tiempo transcurrido entre la exposición y el inicio de los síntomas de la infección ^10^. Según los datos obtenidos en China, en el caso de la COVID-19 es de cinco días en promedio ^12^.

*Periodo prodrómico:* aparición de signos generales inespecíficos. Hay afectación o infección, pero el agente patógeno no actúa sobre los órganos diana ^10^.

*Portador:* persona que no padece los síntomas ni presenta los signos de la enfermedad, pero transmite el agente patógeno ^10^.

*Reservorio:* ser animado o inanimado en el que el agente etiológico se reproduce y perpetúa durante cierto periodo ^8^. En la infección por SARS-CoV-2 se han establecido como posibles animales reservorios los murciélagos y los pangolines, los cuales no presentan los signos de la enfermedad, aunque albergan el virus y lo pueden transmitir a los humanos ^12^.

*Riesgo de contagio:* probabilidad de que ocurra la transmisión de la fuente a un huésped ^10^.

*Tasa de ataque:* forma de incidencia que mide la proporción de personas en una población que experimenta una condición de salud aguda en un periodo limitado. Se calcula como el número de casos nuevos durante el pico dividido por el tamaño de la población al inicio del periodo ^10^.

*Tasa de ataque secundaria:* frecuencia de casos nuevos de la enfermedad entre los contactos de pacientes conocidos ^10^.

*Tasa de contacto:* tasa a la que un individuo infectado infecta a otros sujetos susceptibles a la infección. Esto depende de la exposición e interacción física que tenga el individuo con otras personas vulnerables ^10^. Las medidas de contención y mitigación buscan, entre otras cosas, disminuir esta tasa ^13^^,^^14^.

*Tasa de letalidad:* proporción de personas con una condición particular, que mueren a causa de ella ^10^. Por ejemplo, la tasa de letalidad del dengue grave es de 4,7 % en Colombia ^15^, en tanto que se ha reportado que la de COVID-19 fue de 4 % en China y hasta de 13 % en Italia. Cabe aclarar que esta tasa varía según la detección de casos y el registro de las muertes. En el caso de la COVID-19 en muchos países el registro se limita a los fallecimientos que ocurren en hospitales en personas con prueba confirmatoria de la enfermedad. Se estima un subregistro promedio del 50 % de los casos reales ^12^.

*Tasa de mortalidad:* frecuencia de muerte en una población definida en un periodo. En este caso, la detección de los casos no influye tanto ^10^.

*Transmisibilidad:* capacidad de un material para transmitir fluidos o gérmenes ^8^.

*Transmisión:* mecanismo por el que un agente infeccioso se propaga a un huésped susceptible. Puede ocurrir de diferentes formas: por contacto (incluido el contacto con mucosas), de forma aérea (gotas o aerosoles), y por ingestión o vía placentaria ^8^. En el caso de la COVID-19, la transmisión ocurre por contacto con mucosas y por vía aérea mediante gotas, y de aerosoles al hacer procedimientos médicos como la toma de muestras en orofaringe o la intubación orotraqueal ^16^.

*Vector:* agente que transmite el agente patógeno a otro ser vivo ^8^, por ejemplo, el mosquito *Aedes aegypti,* que transmite el virus del dengue.

*Virus:* agente infeccioso que solo puede reproducirse en tejidos vivos de otros seres. La forma extracelular inerte se llama virión, forma en que penetra a la membrana celular del huésped y libera su ácido nucleico ^8^.

*Zoonosis:* enfermedad o infección que es naturalmente transmisible de animales a humanos, por ejemplo, la COVID-19, la rabia y la toxoplasmosis, entre otras; cerca del 60 % de las infecciones que afectan al ser humano son enfermedades zoonóticas ^8^.

### Ciclo de epidemias y pandemias

En la sección anterior se señalaron algunos factores del agente patógeno y de los huéspedes que determinan la conducta de las enfermedades infecciosas. Asimismo, deben considerarse otras variables como los factores genéticos y biológicos, el ambiente ecológico y físico, el comportamiento humano y demográfico, y los factores sociales, políticos y económicos que, entre otros aspectos, influyen en la propagación de la enfermedad. Las epidemias y las pandemias suelen tener las siguientes fases de evolución que varían según su propagación y las variables mencionadas ^13^.

*Amplificación:* la incidencia de la enfermedad crece a tal punto que cumple con los criterios de epidemia o pandemia. El agente patógeno es capaz de transmitirse de persona a persona y causa un pico sostenido en la comunidad ^13^.

*Inmunidad colectiva:* también conocida como *inmunidad de rebaño,* se refiere al efecto indirecto de la inmunización masiva por infección o vacunación (o su combinación). Se evidencia por la disminución de la infección en personas vulnerables debido a la inmunización de la mayor parte de la población ^13^.

*Introducción:* aparición de un nuevo agente patógeno en la población ^13^.

*Segunda ola:* nuevo brote del mismo agente patógeno que se desarrolla una vez ha disminuido el brote inicial. Dependiendo de la enfermedad puede dar paso a una tercera o cuarta ola hasta que el agente patógeno es erradicado o se hace endémico ^13^.

*Transmisión localizada:* ocurre transmisión esporádica en una localización determinada y con baja incidencia ^13^.

*Transmisión reducida por inmunidad:* disminución de la vía de contagio de persona a persona debido a la adquisición de inmunidad efectiva en la población ^13^.

### Criterios para la adopción de decisiones sobre las medidas de salud pública

*Gravedad y letalidad:* estas condicionan la magnitud del impacto y la aceptación social de las medidas no farmacológicas. La *gravedad* se refiere al grado de compromiso de la enfermedad y de la necesidad de tratamiento y hospitalizacion de las personas enfermas. La *letalidad* se refiere al riesgo de mortalidad que una enfermedad conlleva para el paciente que la sufre ^17^. En el caso de la COVID-19, la OMS ha estimado una tasa de mortalidad, aproximadamente, de 3 % a nivel mundial ^18^.

*Historia natural de la infección:* se refiere al comportamiento de la infección sin medidas de prevención o tratamiento e incluye el periodo de contagio, la incubación, y los estadios prodrómico y clínico. Aporta información sobre las tasas de letalidad y el número reproductivo básico (R_0_), entre otras ^17^.

*Parámetros epidemiológicos:* incluyen el número reproductivo básico (R_0_), el tiempo de generación de la enfermedad, es decir, la velocidad en que se deben activar las medidas de intervención para frenar el crecimiento de la epidemia, y la proporción de la transmisión antes del inicio de los síntomas ^17^^,^^19^.

### Medidas de salud pública ante la pandemia

**Figura 1:**
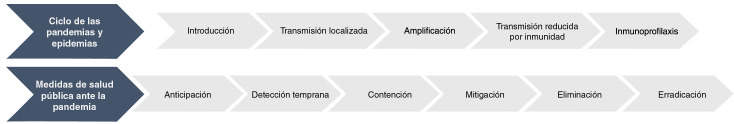
Medidas de salud pública según el ciclo de las epidemias

Dado que no se cuenta con vacunas o tratamientos farmacológicos, y parece que no los habrá pronto, las alternativas restantes son las intervenciones no farmacológicas. Por ello, el objetivo principal de las medidas es impedir o retrasar la diseminación generalizada lo antes posible, inicialmente en el lugar de origen y, posteriormente, en todos los sitios donde se halle presente la pandemia. Con ello se busca disminuir la tasa de incidencia y ganar tiempo para llevar a cabo los planes de respuesta, desarrollar mejores pruebas diagnósticas, disminuir el pico en la demanda de servicios para no sobrecargar el sistema de salud, desarrollar algún tratamiento o inmunoprofilaxis, y preparar a los sectores pertinentes ^17^^,^^20^^,^^21^. Con esta información, y teniendo en cuenta el ciclo de las pandemias (figura 1), se llevan a cabo las siguientes intervenciones de forma secuencial.

*Anticipación:* fortalecimiento de la atención de los factores de riesgo poblacionales y del sistema de salud antes de la aparición de una epidemia. Se pronostican las enfermedades con mayores probabilidades de emerger y se determinan los factores modificadores del efecto de la historia de la enfermedad tanto por su impacto como por su diseminación, y se tienen en cuenta las experiencias pasadas con condiciones similares ^13^.

*Detección temprana:* esta estrategia se lleva a cabo en el ámbito de la salud y se apoya en una vigilancia epidemiológica constante. Se trata de identificar y estudiar la enfermedad, así como sus características asociadas. Debe hacerse coordinadamente con las medidas de contención, ya que busca disminuir la transmisión en la comunidad aislando inicialmente a los pacientes gravemente enfermos. Una vez se surte esta etapa se puede proceder con las otras medidas necesarias para el manejo de la epidemia ^13^.

*Contención:* fase fundamental para evitar la epidemia a gran escala; debe iniciarse tan pronto como se detecta el primer caso, independientemente de su etiología y origen.

Entre sus estrategias se encuentran ^13^^,^^14^:

- la higiene individual y el control hospitalario de la infección mediante el lavado de manos, la desinfección de implementos (médicos y de uso diario), y el uso de equipamiento de protección personal según el mecanismo de transmisión del patógeno; en los contextos clínicos, se incluye el adecuado etiquetamiento y disposición de residuos;
- el distanciamiento social (físico) y evitar eventos públicos masivos;
- el cierre de centros educativos y el fomento de teletrabajo;
- la atención a las necesidades de la población para evitar que salga a espacios públicos como mercados, tiendas y farmacias, con apoyo especial para las poblaciones vulnerables (mayores de 70 años, pacientes con cormibilidades);
- los controles internacionales en las fronteras terrestres, aéreas y marítimas, con opciones que van desde las tamizaciones a la entrada y salida de las personas hasta el cierre de las fronteras;
- el aislamiento de las personas infectadas y la cuarentena de los casos sospechosos, y
- el entrenamiento y la preparación antes del pico de la enfermedad para los profesionales e instituciones que enfrentan el reto, así como de los sistemas que soportan la carga, con lineamientos centrados en la enfermedad emergente.

*Control y mitigación:* intervención que se aplica cuando la infección en la población alcanza la fase de epidemia o pandemia. El objetivo es proveer cuidado oportuno y de calidad a los pacientes que lo necesiten, mitigar o reducir el impacto de la enfermedad y reducir su incidencia, morbimortalidad, así como las alteraciones secundarias de los sistemas económicos, sociales y políticos.

Para lograr una mitigación de la infección hay diferentes alternativas, entre ellas ^11^^,^^13^^,^^14^^,^^22^^,^^23^:

- el uso de vacunas y medicamentos para evitar el contagio y tratar los casos que ya tienen la enfermedad (esta alternativa no está disponible en la infección por SARS CoV2);
- ganar tiempo y permitir el desarrollo de nuevas tecnologías de prevención, diagnóstico y tratamiento;
- reducir la diseminación de la enfermedad con base en la utilización y adaptación de las medidas utilizadas en la contención, pero con una intensidad mayor, que pueden ir desde el fomento del teletrabajo hasta decretar una cuarentena de la población;
- aumentar el cuidado y la vigilancia de cada caso para disminuir la gravedad de la enfermedad y la probabilidad de contagio;
- aumentar la cobertura de la atención de los casos y la capacidad diagnóstica y, si es necesario, realizar vigilancia activa en la comunidad, y
- prestar especial atención a los comunicados y la información que se difunde a la población.

*Acordeón:* se ha recurrido a este termino coloquial para referirse a una estrategia de manejo epidémico a largo plazo caracterizada por el establecimiento de medidas de control y su posterior liberación hasta que existan procedimientos de inmunización exitosos (vacuna o exposición de la mayoría de la población) o tratamientos efectivos ^24^.

*Eliminación:* es la siguiente fase, la cual implica la disminución masiva de casos activos e incidentes; en ella se declara que ya no hay epidemia (como en Nueva Zelanda durante la primera semana de mayo). En esta fase, la enfermedad ya no se considera un problema de salud pública, aunque deben mantenerse las medidas de vigilancia y control para prevenir su reaparición ^13^.

*Erradicación:* eliminación permanente de la incidencia a nivel mundial; ya no se necesitan medidas adicionales, pues se considera que no hay riesgo de reaparición. Una enfermedad se considera erradicada cuando se cumplen estos tres criterios: se cuenta con una intervención disponible para interrumpir su transmisión; se tienen herramientas diagnósticas eficientes para detectar casos, y los humanos son los únicos reservorios ^13^.

### Estrategias no farmacológicas en la comunidad

Las estrategias no farmacológicas en la comunidad deben aplicarse preferiblemente antes del pico de la infección e ir acompañadas de las siguientes intervenciones para predecir el comportamiento y prevenir la propagación de la infección.

*Aislamiento:* separación de las personas infectadas o sintomáticas de las personas que no lo están para evitar la transmisión a los individuos susceptibles ^25^.

*Control de la infección:* medidas de higiene y protección para reducir el riesgo de transmisión de un agente infeccioso de una persona a otra, entre ellas, el lavado de manos y el uso de tapabocas en personas sintomáticas o con diagnóstico confirmado ^11^.

*Cuarentena:* término que se originó en Venecia durante la epidemia de peste bubónica en 1370; se refiere al número de días (cuarenta) que los barcos debían permanecer en el agua antes de que desembarcara su tripulación y se descargaran sus mercancías. Es una medida preventiva que consiste en la separación de personas sanas que han estado expuestas -o que se sospecha que lo han estado- a una enfermedad transmisible para monitorear su evolución y prevenir la potencial transmisión ^11^^,^^22^^,^^26^.

*Distanciamiento social:* aumentar el espacio y evitar el contacto físico entre dos personas para evitar la propagación de una enfermedad. En el caso de la infección por SARS-CoV-2 se sugiere una distancia de dos metros entre personas para eludir el contagio por la dispersión de gotas y aerosoles. También incluye medidas como el teletrabajo, el cierre de escuelas y la cancelación o postergación de reuniones con grandes cantidades de personas ^25^.

*Intervención contra la fatiga:* comportamientos que optimicen la salud física y mental, o desalienten comportamientos potencialmente adversos para la salud o el control de la infección ^11^.

*Modelado:* simulación de situaciones de la vida real con ecuaciones matemáticas para aproximarse a sucesos futuros basada en experiencias pasadas con el mismo agente patógeno o con agentes de los cuales se suponga un comportamiento similar. Se utiliza para ilustrar circunstancias posibles y facilitar la preparación y la adopción de decisiones ^27^.

*Vigilancia:* es la observación continua y cercana de una o más personas o de la población en general con un fin de control o supervisión; se refiere al seguimiento de los problemas de salud pública, con el fin de recolectar la información necesaria para entender el problema epidémico e informar sobre él. Provee información oportuna, describe las características de la transmisión y las medidas que pueden ser tomadas para evitar su diseminación. Su metodología incluye el diagnóstico rápido, la tamización, la notificación y el reporte de casos, la investigación de los contactos y el monitoreo de tendencias ^11^^,^^22^.

### Comentario

Las decisiones en salud pública y las indicaciones para los profesionales de la salud y la población en general recurren a muchos de los términos aquí expuestos. De ello se desprende la importancia de la literatura especializada mediante la cual es posible obtener, procesar y entender la información básica de salud y las herramientas para la adopción de decisiones ^28^^,^^29^. La literatura especializada en salud se centra en el paciente y supone una interacción entre todas las partes involucradas en el sistema de salud (pacientes, personal clínico y administrativo).

Debe tenerse en cuenta que no se trata aquí de conceptos unidimensionales, por lo que deben considerarse distintos componentes como el contexto del servicio de salud o del paciente, las creencias y costumbres culturales, los mitos referentes a la salud, etc., que influyen en la cognición de los individuos.

En otras palabras, la literatura especializada en salud va más allá de la simple información o de las indicaciones ^28^^-^^30^, y no olvidarlo resulta fundamental en el manejo de los problemas en salud, sobre todo si causan incertidumbre y miedo como en el caso de una pandemia ^31^, situación que entraña el reto de la comunicación y recalca la importancia de conocer los términos utilizados por los profesionales de la salud, especialmente cuando se trata de un agente patógeno nuevo y no se conoce todo sobre él, por lo que se corre el riesgo de dar información ambigua. Se ha podido comprobar que cuando los mensajes se transmiten de esta manera se reduce el nivel de cumplimiento de las medidas de prevención y de tratamiento incluso más que cuando se admite que no se conoce cabalmente la enfermedad ^32^^,^^33^.

Si el mensaje se transmite adecuadamente, debe incentivarse la consulta de la literatura especializada en salud entre la población, fomentando así su participación activa a nivel individual y comunitario. Dicha participación se ha asociado con mejores resultados en salud, con una mayor calidad de vida y con la disminución de los síntomas psicosomáticos y las conductas de riesgo, incluido el consumo de sustancias psicoactivas o la desestimación de las medidas preventivas ^28^^-^^30^^,^^33^. Es por ello que se presenta en este artículo un ABC homogéneo de los conceptos sobre el coronavirus, resaltando la importancia de las medidas vigentes para el control de la enfermedad y estableciendo los conceptos básicos de las enfermedades infecciosas, aspectos claves para el manejo de la actual pandemia y de las que eventualmente ocurran en el futuro.
